# Adrenal cortical neoplasms: a study of clinicopathological features related to epidermal growth factor receptor gene status

**DOI:** 10.1186/1746-1596-9-19

**Published:** 2014-01-23

**Authors:** Jing Zhang, Cuiping Wang, Jie Gao, Jian Sun, Xuan Zeng, Shafei Wu, Zhiyong Liang

**Affiliations:** 1 Department of Pathology, Peking Union Medical College Hospital, Chinese Academy of Medical Sciences and Peking Union Medical College, 1 Shuai Fu Yuan Hu Tong, Beijing 100730, People’s Republic of China

**Keywords:** Adrenal cortical neoplasm, Epidermal growth factor receptor, Fluorescence in situ hybridization, Mutation

## Abstract

**Background:**

Adrenocortical carcinoma (ACC) is a rare but highly malignant neoplasm with limited treatment options.

**Methods:**

In this study, the clinicopathological features of 22 ACCs and 22 adrenocortical adenomas (ACA) were analyzed, and the EGFR protein expression, EGFR gene mutation status and EGFR gene copy number alteration of all tumors were examined using immunohistochemistry, fluorescence in situ hybridization (FISH), and the Scorpion Amplification Refractory Mutation System (ARMS), respectively.

**Results:**

EGFR protein expression was detected in 63.6% of the ACC samples, and EGFR FISH was positive in 50% of the ACC samples (all were high polysomy on chromosome 7). In contrast, 27.3% of the ACA samples demonstrated EGFR expression, and none of the ACA samples tested positive by FISH. There were significant differences between the ACC and ACA in terms of protein expression (P = 0.035) and gene copy number alterations (P < 0.001).

**Conclusions:**

EGFR protein expression and high polysomy on chromosome 7 are frequent abnormalities in ACC than in ACA.

**Virtual slides:**

The virtual slide(s) for this article can be found here: http://www.diagnosticpathology.diagnomx.eu/vs/2068470757103500.

## Background

Adrenocortical tumors are a common type of tumor and have a prevalence of at least 3% in populations over the age of 50 [1]. The vast majority of these tumors are benign adrenocortical adenomas (ACA), and only a small subset constitutes malignant adrenocortical carcinomas (ACC) [2]. These tumor entities are discriminated according to gross and microscopic criteria, such as the Weiss score [3]. Although each criterion is strictly defined, some features are potentially more problematic (e.g., diffuse architecture, necrosis, and sinusoidal, venous and capsular invasions). However, thus far, no generally accepted marker has been established to determine malignancy in these tumors [4,5]. ACC is a rare endocrine tumor with a poor overall prognosis [1]. Complete surgical resection is the only available curative treatment, and distant recurrences are common even after these procedures [6]. The genetic background of ACC is poorly understood, and further study is necessary to identify novel targets for tailored therapies.

It has been proposed that the epidermal growth factor receptor (EGFR) pathway is important for cancer pathophysiology. EGFR belongs to the ErbB family of tyrosine kinase receptors, which have strong regulatory effects on cell proliferation, differentiation, survival, and migration [7]. Increased levels of EGFR gene expression have been reported in adenocarcinomas of the head and neck, lung, pancreas, bladder, ovary, cervix, brain, breast, colon and prostate, among others, and have been frequently associated with adverse prognoses [7-9]. In recent years, EGFR has become a promising target for therapies against various tumor entities. For example, treatment with the monoclonal anti-EGFR antibody cetuximab and the EGFR tyrosine kinase inhibitor erlotinib (in monotherapy or in combination with cytotoxic drugs) has resulted in improved survival in patients with colorectal cancer and non-small-cell lung cancer [10,11].

For ACCs, EGFR expression and *EGFR* point mutations have also been described [12-16]. However, there has been no comprehensive investigation of EGFR protein expression or somatic EGFR gene mutations and amplifications for adrenocortical neoplasms. In our previous study, we have found that EGFR protein expression was more frequent in myxoid ACC than in myxoid ACA [17]. However, *EGFR* mutations and EGFR amplification were rare in these myxoid tumors. In this study, we analyzed the clinicopathological features of 22 conventional ACC and 22 conventional ACA samples. Furthermore, we explored EGFR protein expression, *EGFR* mutations and EGFR copy number in these tumors and correlated these results with the clinicopathological features of these patients.

## Materials and methods

### Case selection

A total of 1355 consecutive adrenocortical tumors that were surgically resected at the Department of Surgery at Peking Union Medical College Hospital during the period between 2000 and 2012 were reviewed, and specimens were selected from archived paraffin-embedded blocks. Among these tumors, there were 22 cases of conventional ACC. At the same time, 22 cases of conventional ACA were randomly selected. (including 5 cases of conventional ACC and 5 cases of conventional ACA which have been published before [17]). The diagnoses were based on clinical, biochemical and morphological data. All histological diagnoses were confirmed by two pathologists at the Peking Union Medical College Hospital. Clinicopathological information was obtained by reviewing the medical records in detail with regard to patient age and sex, the date of surgery, the pathological stage and the histological findings. The follow-up period ranged from 2 to 99 months, with a mean of 28.3 months for all patients. This study was conducted with the approval of the Ethics Committee of the Peking Union Medical College Hospital and with the informed consent of all patients.

### Immunohistochemical study

EGFR protein expression was evaluated by immunohistochemistry using a mouse anti-human EGFR monoclonal antibody (clone 2-18C9, Pharm Dx kit, Dako North America, Inc., Via Real, Carpinteria, CA, USA), according to the manufacturer’s instructions. Sections (4 μm thick) were cut from paraffin tissue blocks, deparaffinized in xylene, and rehydrated in decreasing concentrations of ethanol. Appropriate positive and negative controls were used, and positive results were defined as samples demonstrating greater than 10% of tumor cells with membranous staining of any intensity. The percentage of positive cells and the intensity, which was defined as mild (1+), moderate (2+) or strong (3+) were recorded for each sample.

### DNA extraction and *EGFR* mutations using the scorpion amplified refractory mutation system

Formalin-fixed, paraffin-embedded tumor sections were deparaffinized and air-dried. Tumor cells were acquired by micro-dissection from 6–8 slides of each sample. Genomic DNA from the tumors was extracted with standard proteinase K digestion and a DNeasy minispin column (TIANamp Genomic DNA Kit, BEIJING). Twenty-nine specific *EGFR* mutations in exons 18 through 21 were detected using the EGFR mutation test kit of the Scorpion Amplified Refractory Mutation System (SARMS) (DxS, Manchester, UK). This kit enabled the detection of 19 specific deletions between 2235 and 2257 in exon 19, T790M, L858R, L861Q, G719X, S768I as well as 3 insertions in exon 20. The mutant assays, which used allele-specific real-time polymerase chain reactions, were performed according to the manufacturer’s protocol using the Applied Biosystem (ABI) 7500 real-time PCR system [18-20]. The data were analyzed using the ABI SDS software.

### Fluorescent *in situ* hybridization

An EGFR FISH analysis was performed using the LSI EGFR SpectrumOrange/CEP 7 SpectrumGreen probe (Vysis, Abbott Laboratories), according to the manufacturer’s protocol. The FISH analyses were performed independently by two authors who were blinded to the clinical characteristics of the patients and to all other molecular variables. For the EGFR FISH analyses, 60 nuclei were scored for signals from both DNA probes using an Olympus BX51TRF microscope (Olympus, Japan) equipped with a triple-pass filter (DAPI/Green/Orange; Vysis) at a final magnification of 1000 × .

Chromosome 7 polysomy and monosomy were defined as the presence of more than three signals or one signal, respectively, from greater than 20% of the tumor cells. The EGFR gene status was classified into the following six categories according to the frequency of tumor cells with specific copy numbers of the EGFR gene and the chromosome 7 centromere, as described previously [21]. Based on the EGFR gene status, the patients were further classified into the following two groups: 1) EGFR FISH-negative or low gene copy number (disomy, low trisomy, high trisomy, and low polysomy) or 2) EGFR FISH-positive or high gene copy number (high polysomy and gene amplification). For each FISH preparation, known positive and negative cells were used as controls.

### Statistical analysis

Correlations between the different groups were evaluated by Pearson’s Chi-squared test or Fisher’s two-tailed exact test. The mean values of continuous data were assessed using independent samples *t* test. Survival curves were plotted using the Kaplan-Meier method, and P-values were calculated using the log-rank test. Data were analyzed with SPSS software for Windows, version 15.0 (SPSS Inc, Chicago, IL). P-values less than 0.05 were accepted as significant.

## Results

### Clinical data

Table 1 summarizes the clinical data for both malignant and benign adrenocortical tumors. The ages of patients ranged from 2 to 75 years, with mean age of 45.4 years. The overall male/female ratio was 1:1. Fifteen patients had hormonal abnormalities (7 with an ACA and 8 with an ACC), 9 patients had Cushing syndrome (3 with an ACA and 6 with an ACC), 5 had primary hyperaldosteronism (4 with an ACA and 1 with an ACC), and 1 male ACC patient with concomitant feminization. None of the patients had a prior history of malignancy. There were no significant differences in terms of patient age or sex, the existence of endocrine syndrome, or the tumor location between the malignant and benign tumor samples. Of the patients with malignant tumors, 6 demonstrated synchronous metastasis, and 11 experienced tumor recurrence (local recurrence and/or metachronous distant metastasis) during the follow-up period. Twelve patients died from the disease (mean overall survival was 19.6 months), 2 were alive with the disease, 3 were alive without the disease at the final follow-up, and 5 were lost track of during follow-up. Of the patients with benign tumors (mean follow-up of 25.5 months), 16 were alive without disease following the operation, and 6 were lost track of during follow-up. The malignant tumors had a significantly larger size and weight than the benign tumors (P < 0.001) (Table 1).

**Table 1:** Clinicopathological features and EGFR gene status of the 44 adrenocortical tumors

	**Malignant (n = 22)**	**Benign (n = 22)**	**p value**
Age (yrs)			
Mean	43.4	47.4	0.364
Range	2–75	34–69	
Sex			
Males	13	9	0.228
Females	9	13	
Hormonal function			
Functioning	8	7	0.498
Non-functioning	14	15	
Tumor location			
Right	14	13	0.757
Left	8	9	
Tumor size (cm)			
Mean	12.3	2.9	<0.001^*^
Range	3-24	0.8-5.5	
Tumor weight (g)			
Mean	847.0	10.6	<0.001^*^
Range	12-3250	1-42	
EGFR copy number			
Positive	11	0	<0.001^*,†^
Negative	11	22	
EGFR IHC			
Positive	14	7	0.035^*^
Negative	8	15	

EGFR, epidermal growth factor receptor; IHC, immunohistochemistry.

*Statistically significant.

^†^Fisher’s exact test (two-tailed).

### EGFR protein expression in adrenocortical tumors

**Figure 1:**
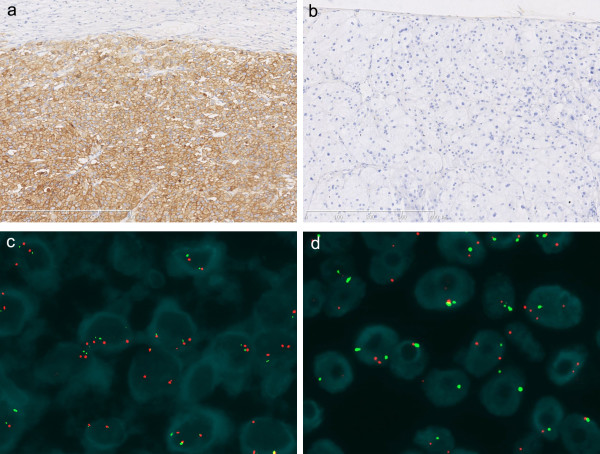
**EGFR (epidermal growth factor receptor) gene status in adrenocortical neoplasms: (a) This image demonstrates strong membrane EGFR expression (3+) in adrenocortical carcinoma, as detected by immunohistochemistry. (b)** This image demonstrates an absence of membrane EGFR expression in adrenocortical adenoma, as detected by immunohistochemistry. **(c)** The cancer cells demonstrate high polysomy on chromosome 7 in adrenocortical carcinoma, as detected by FISH(fluorescence in situ hybridization). **(d)** The tumor cells display disomy for the EGFR in adrenocortical adenoma, as detected by FISH (Green signals represent the chromosome 7 centromere, and red signals represent the EGFR gene).

Overall, EGFR expression was detected in 14 ACC cases (63.6%), including 6 samples where the tumor cells showed strong staining intensities (3+) (Figure 1a). In addition, EGFR immunoreactivity was moderate (2+) in 3 cases and weak (1+) in 5 cases, whereas no specific staining was detected in 8 cases (36.4%). In contrast, 7 ACA cases (31.8%) showed EGFR expression; none demonstrated 3+ EGFR immunoreactivity, 2 cases showed 2+ EGFR levels and 5 cases showed 1+ EGFR levels. The remaining 15 cases (68.2%) showed level 0 staining (Figure 1b). There was a statistically significant difference in the EGFR immunostaining between benign and malignant tumors (P = 0.035) (Table 1). Among the 22 ACC cases, EGFR protein expression was not associated with gender, age, tumor size, tumor weight, hormonal function, recurrence, metastasis or tumor stage (Table 2).

**Table 2:** Relationship between EGFR protein expression, EGFR copy number and clinicopathological characteristics of adrenocortical carcinomas

	**FISH positive (n = 11)**	**FISH negative (n = 11)**	**P-value**	**IHC positive (n = 14)**	**IHC negative (n = 8)**	**P-value**
Sex						
Males	7	6	1.000^*^	8	5	1.000^*^
Females	4	5		6	3	
Age (yrs)						
Mean	38.6	48.2	0.224	42.8	44.5	0.836
Range	2-61	20-75		2-75	17-72	
Tumor size (cm)						
Mean	11.5	13.0	0.594	10.5	15.3	0.097
Range	3-24	4-24		3-24	7-24	
Tumor weight (g)						
Mean	761.8	932.1	0.730	579.9	1314.4	0.102
Range	12-2700	16-3250		12-3250	95-2700	
Hormonal function						
Functioning	5	3	0.659^*^	7	1	0.079^*^
Non-functioning	6	8		7	7	
Recurrence or metastasis						
Yes	5	6	0.670	6	5	0.659^*^
No	6	5		8	3	
TNM stage						
I	1	0	1.000^*^	1	0	1.000^*^
II-IV	10	11		13	8	

EGFR, epidermal growth factor receptor; IHC, immunohistochemistry; FISH, fluorescence in situ hybridization.

^*^Fisher’s exact test (two-tailed).

### *EGFR* mutations and copy number in adrenocortical tumors

None of the 29 specific *EGFR* mutations in exons 18 to 21 were detected in the ACA and ACC cases examined.

None of the ACC cases demonstrated EGFR gene amplification, 11 cases showed high polysomy (Figure 1c), 5 cases showed low trisomy, 6 cases showed disomy and no cases displayed evidence of low polysomy or high trisomy. None of the ACA cases demonstrated EGFR gene amplification, although there were also no ACA cases with high polysomy or high trisomy. Two ACA cases showed low polysomy, 6 casesshowed low trisomy, and 14 cases showed disomy (Figure 1d). According to the criteria of Capuzzo et al. [21], eleven ACC cases (50%) showed FISH positivity, and all ACA cases demonstrated FISH negativity. There was a statistically significant difference in the EGFR gene copy number alterations between the benign and malignant tumors (P < 0.001) (Table 1). EGFR FISH positivity was not found to be associated with gender, age, tumor size, tumor weight, hormonal function, recurrence, metastasis or tumor stage (Table 2).

### Relationship between EGFR protein expression, EGFR copy number and clinicopathological characteristics of ACC

Among the 22 ACC cases, EGFR protein expression was not associated with EGFR FISH positivity (Table 3). Nine of the 11 (81.8%) EGFR FISH-positive cases,all of which were classified as high polysomy, showed EGFR protein expression. Five of the 11 (45.5%) FISH-negative cases, which included 2 low-trisomy cases and 3 disomy cases, showed EGFR protein expression. And 5 EGFR protein expression cases were FISH-negative. The survival curves that were derived using the Kaplan–Meyer method demonstrated that there was no significant association between EGFR protein expression and the overall survival of patients with ACC. The survival curves also demonstrated that there was no significant association between high polysomy on chromosome 7 and the overall survival of patients with ACC.

**Table 3:** Relationship between EGFR copy number and EGFR protein expression of in adrenocortical carcinomas

**IHC**	**FISH**	
	**Positive**	**Negative**
Positive	9	5
Negative	2	6
P value	0.183	

EGFR, epidermal growth factor receptor; IHC, immunohistochemistry; FISH, fluorescence in situ hybridization.

## Discussion

The molecular changes that are involved in the pathogenesis of ACC are poorly understood. TNM classification remains the most commonly used parameter for guiding therapy and prognosis. However, there is a need for new prognostic and therapeutic markers, particularly with the development of new molecular-targeted therapies, including anti-EGFR molecules. In this study, we have investigated EGFR protein expression, *EGFR* mutations, and EGFR gene copy number variations in adrenocortical neoplasms, specifically in 22 conventional ACC cases and 22 conventional ACA cases.

EGFR is a member of the EGF-related family of tyrosine kinase receptors. Upon the binding of EGFR ligands, downstream signaling pathways are activated, which results in strong stimulatory effects on cell proliferation, differentiation, survival, angiogenesis and migration [7,8]. The expression of EGFR in ACC has been described in previous studies [12-15]. However, some of these studies have not differentiated between cytoplasmic and membranous staining, and only the latter type is currently considered to represent specific staining. In our previous study, we have found that EGFR protein expression was more frequent in myxoid ACC than in myxoid ACA [17]. In the current study, our results also demonstrated that EGFR immunoreactivity was significantly more frequent among conventional ACC than conventional ACA cases. The relative abundance of EGFR in ACC suggests that anti-EGFR agents may be beneficial for patients with ACC.

Cetuximab, gefitinib, and erlotinib, chemotherapeutic agents that target the EGFR gene, have been administered to patients with cancer (e.g., non-small-cell lung cancer) [22]. In addition, EGFR gene mutations have been reported in patients with non-small-cell lung cancer, and the status of these mutations has been correlated with the clinical response to tyrosine kinase inhibitors such as gefitinib [23]. Kotoula et al. detected four tumor specimens that harbored TK domain mutations from a panel of 35 ACC (11.4%), suggesting that ACC that harbor an *EGFR* mutation exhibit increased phosphorylation of EGFR compared with wild-type carcinomas [16]. In contrast, our previous and current study did not detect any mutations in samples of ACC and ACA [17]. It is possible that the method used in this study was only able to detect 29 of the most common mutations.

EGFR gene amplification and structural genetic alterations have been reported in several types of adenocarcinoma, including non-small-cell lung cancer, glioblastoma, pancreatic cancer, and squamous cell carcinoma of the head and neck.

In our previous study of 10 myxoid adrenocortical neoplasms, only 1 ACA showed high polysomy on chromosome 7 [17]. In current study, we examined the EGFR gene copy number in conventional ACC and ACA. Moreover, our study analyzed the relationship between EGFR copy number and the clinicopathological features of patients with ACC. In our series of cases, 50% of the ACC cases showed FISH positivity, and all ACA cases demonstrated FISH negativity based on the criteria of Capuzzo et al. [21]. We discovered that there was a statistically significant difference between benign and malignant tumors regarding their alterations in the EGFR gene copy number. This result could potentially be used for the differential diagnosis of ACA and ACC. In addition, all of the positive cases showed high polysomy on chromosome 7, and none of the ACC cases demonstrated EGFR amplification, suggesting that EGFR amplification may be rare in ACC. However, according to the statistical analysis, EGFR FISH positivity was not associated with gender, age, tumor size, tumor weight, hormonal function, recurrence, metastasis or tumor stage. Therefore, our study does not provide evidence that high polysomy is related to the aggressive nature of these tumors. The survival curves demonstrated that there were no significant associations between EGFR protein expression, high polysomy on chromosome 7 and the overall survival of patients with ACC. This result may be related to certain cases that were lost track of during follow-up, but it would be necessary to increase the number of cases and the rate of follow-up for future studies.

In our study, EGFR protein expression and high polysomy were more frequently seen in conventional ACC than in conventional ACA, and EGFR immunohistochemical staining were more intensive in ACC cases than in ACA cases, whether this can be used as differential diagnosis still need to be demonstrated by large sample size. Furthermore, no association was found between EGFR protein expression and alterations in the EGFR gene copy number, this suggested that EGFR protein expression may not be due to increased copy number, other mechanisms, possibly transcriptional, post-transcriptional or epigenetic, may be associated with EGFR protein expression.

In summary, EGFR expression and high polysomy on chromosome 7 are frequent abnormalities in ACC than in ACA, suggesting that these abnormalities could potentially be used in the differential diagnosis of ACA and ACC. Furthermore, an investigation of the EGFR gene status may provide further information regarding potential therapeutic targets in patients with ACC.

## Competing interests

The authors declare that they have no competing interests.

## Authors’ contributions

JZ designed the experiment and drafted the manuscript, CW immunohistochemistry staining and FISH, JG performed mutational detection, JS designed the experiment and statistically analysis, XZ and SW performed FISH analysis, ZL conceived of the study, and participated in its design and coordination. All authors read and approved the final manuscript.

## References

[B1] Allolio B Fassnacht M Clinical review: adrenocortical carcinoma: clinical update J Clin Endocrinol Metab 2006 91 2027 2037 10.1210/jc.2005-2639 16551738

[B2] Kuruba R Gallagher SF Current management of adrenal tumors Curr Opin Oncol 2008 20 34 46 10.1097/CCO.0b013e3282f301fd 18043254

[B3] Weiss LM Medeiros LJ Vickery AL Jr Pathologic features of prognostic significance in adrenocortical carcinoma Am J Surg Pathol 1989 13 202 206 10.1097/00000478-198903000-00004 2919718

[B4] Sasano H Suzuki T Moriya T Recent advances in histopathology and immunohistochemistry of adrenocortical carcinoma Endocr Pathol 2006 17 345 354 10.1007/s12022-006-0006-0 17525483

[B5] Volante M Buttigliero C Greco E Berruti A Papotti M Pathological and molecular features of adrenocortical carcinoma: an update J Clin Pathol 2008 61 787 793 10.1136/jcp.2007.050625 18430754

[B6] Libe R Fratticci A Bertherat J Adrenocortical cancer: pathophysiology and clinical management Endocr Relat Cancer 2007 14 13 28 10.1677/erc.1.01130 17395972

[B7] Normanno N De Luca A Bianco C Strizzi L Mancino M Maiello MR Carotenuto A De Feo G Caponigro F Salomon DS Epidermal growth factor receptor (EGFR) signaling in cancer Gene 2006 366 2 16 10.1016/j.gene.2005.10.018 16377102

[B8] Sibilia M Kroismayr R Lichtenberger BM Natarajan A Hecking M Holcmann M The epidermal growth factor receptor: from development to tumorigenesis Differentiation 2007 75 770 787 10.1111/j.1432-0436.2007.00238.x 17999740

[B9] Sharma SV Bell DW Settleman J Haber DA Epidermal growth factor receptor mutations in lung cancer Nat Rev Cancer 2007 7 169 181 10.1038/nrc2088 17318210

[B10] Cunningham D Humblet Y Siena S Khayat D Bleiberg H Santoro A Bets D Mueser M Harstrick A Verslype C Chau I Van Cutsem E Cetuximab monotherapy and cetuximab plus irinotecan in irinotecan- refractory metastatic colorectal cancer N Engl J Med 2004 351 337 345 10.1056/NEJMoa033025 15269313

[B11] Shepherd FA Rodrigues Pereira J Ciuleanu T Tan EH Hirsh V Thongprasert S Campos D Maoleekoonpiroj S Smylie M Martins R van Kooten M Dediu M Findlay B Tu D Johnston D Bezjak A Clark G Santabárbara P Seymour L National Cancer Institute of Canada Clinical Trials Group:Erlotinib in previously treated non-small-cell lung cancer N Engl J Med 2005 353 123 132 10.1056/NEJMoa050753 16014882

[B12] Sasano H Suzuki T Shizawa S Kato K Nagura H Transforming growth factor alpha, epidermal growth factor, and epidermal growth factor receptor expression in normal and diseased human adrenal cortex by immunohistochemistry and in situ hybridization Mod Pathol 1994 7 741 774 7824507

[B13] Kamio T Shigematsu K Sou H Kawai K Tsuchiyama H Immunohistochemical expression of epidermal growth factor receptors in human adrenocortical carcinoma Hum Pathol 1990 21 277 282 10.1016/0046-8177(90)90227-V 2312105

[B14] Nakamura M Miki Y Akahira J Morimoto R Satoh F Ishidoya S Arai Y Suzuki T Hayashi Y Sasano H An analysis of potential surrogate markers of target-specific therapy in archival materials of adrenocortical carcinoma Endocr Pathol 2009 20 17 23 10.1007/s12022-009-9058-2 19184558

[B15] Adam P Hahner S Hartmann M Heinrich B Quinkler M Willenberg HS Saeger W Sbiera S Schmull S Voelker HU Ströbel P Allolio B Fassnacht M Epidermal growth factor receptor in adrenocortical tumors: analysis of gene sequence, protein expression and correlation with clinical outcome Mod Pathol 2010 23 1596 1604 10.1038/modpathol.2010.153 20693985

[B16] Kotoula V Sozopoulos E Litsiou H Fanourakis G Koletsa T Voutsinas G Tseleni-Balafouta S Mitsiades CS Wellmann A Mitsiades N Mutational analysis of the BRAF, RAS and EGFR genes in human adrenocortical carcinomas Endocr Relat Cancer 2009 16 565 572 10.1677/ERC-08-0101 19190079

[B17] Zhang J Sun J Liang Z Gao J Zeng X Liu T Myxoid Adrenocortical Neoplasms A Study of the Clinicopathologic Features and EGFR Gene Status of Ten Chinese Cases Am J Clin Pathol 2011 136 783 792 10.1309/AJCP7LO3NAYQKASZ 22031318

[B18] Newton CR Graham A Heptinstall LE Powell SJ Summers C Kalsheker N Smith JC Markham AF Analysis of any point mutation in DNA: The amplification refractory mutation system (ARMS) Nucleic Acids Res 1989 17 2503 2516 10.1093/nar/17.7.2503 2785681 PMC317639

[B19] Thelwell N Millington S Solinas A Booth J Brown T Mode of action and application of scorpion primers to mutation detection Nucleic Acids Res 2000 28 3752 3761 10.1093/nar/28.19.3752 11000267 PMC110766

[B20] Whitcombe D Theaker J Guy SP Brown T Little S Detection of PCR products using self-probing amplicons and fluorescence Nat Biotechnol 1999 17 804 807 10.1038/11751 10429248

[B21] Cappuzzo F Hirsch FR Rossi E Bartolini S Ceresoli GL Bemis L Haney J Witta S Danenberg K Domenichini I Ludovini V Magrini E Gregorc V Doglioni C Sidoni A Tonato M Franklin WA Crino L Bunn PA Jr Varella-Garcia M Epidermal growth factor receptor gene and protein and gefitinib sensitivity in non-small-cell lung cancer J Natl Cancer Inst 2005 97 643 655 10.1093/jnci/dji112 15870435

[B22] Thatcher N The place of targeted therapy in the patient management of non-small cell lung cancer Lung Cancer 2007 57 Suppl 2 S18 S23 17686441 10.1016/S0169-5002(07)70423-3

[B23] Loprevite M Tiseo M Chiaramondia M Capelletti M Bozzetti C Bortesi B Naldi N Nizzoli R Dadati P Kunkl A Zennaro D Lagrasta C Campanini N Spiritelli E Camisa R Grossi F Rindi G Franciosi V Ardizzoni A Buccal mucosa cells as in vivo model to evaluate gefitinib activity in patients with advanced non small cell lung cancer Clin Cancer Res 2007 13 6518 6526 10.1158/1078-0432.CCR-07-0805 17975165

